# Traits Underlying Experimentally Evolved Dispersal Behavior in *Tribolium castaneum*

**DOI:** 10.1007/s10905-024-09862-x

**Published:** 2024-09-27

**Authors:** Michael D. Pointer, Lewis G. Spurgin, Ramakrishnan Vasudeva, Mark McMullan, Simon Butler, David S. Richardson

**Affiliations:** 1 University of East Anglia, Norwich Research Park, Norwich, NR47TJ UK; 2 Earlham Institute, Norwich Research Park, Norwich, NR4 7UZ UK

**Keywords:** Artificial selection, Boldness, Dispersal, Emigration, Experimental evolution, Flour beetle, Tenebrionidae, *Tribolium*

## Abstract

Dispersal is an important behavior in many animals, with profound effects on individual fitness and the evolutionary trajectories of populations. This is especially true within taxa with particular life-history strategies, for example those that exploit ephemeral habitat. Further, dispersal is commonly seen to be part of behavioral syndromes - suites of traits that covary across behavioral contexts. The red flour beetle, *Tribolium castaneum* (Coleoptera, Tenebrionidae), is a major post-harvest crop pest responsible for large losses through the infestation of stored grain. In this system dispersal is known to have a strong genetic basis and differential artificial selection on dispersal traits produces strong phenotypic divergence. However, it is unknown which traits are able to rapidly evolve to produce these results, or which behavioral components underlie differences in dispersal. Using replicate lines of *T. castaneum* previously selected for divergent dispersal behavior, we test for correlated activity and movement patterns, morphology and substrate surface use. We find robustly repeatable associations between the dispersal phenotype and higher activity, straighter paths, larger body size (but not relative leg length) and increased tendency to remain at the surface of fodder. Together our results suggest that dispersal is part of a syndrome of traits in *T. castaneum*, and must be treated as such when considering the evolution of dispersal in this system, and in attempting to predict and control its spread.

**Supplementary Information:**

The online version contains supplementary material available at 10.1007/s10905-024-09862-x.

## Introduction

Dispersal is a life-history trait with great importance in the ecology and evolution of many species, and across different levels of organisation (Clobert et al. 2012). For the individual, the fitness consequences of relocation to a new environment can be enormous (Clobert et al. 2012), while individual dispersal outcomes aggregate to determine species’ ranges and metapopulation structures through effects on gene flow (Kokko and Lopez-Sepulcre 2006; Ronce 2007). Consequently dispersal is a key parameter underlying evolutionary trajectories and metapopulation persistence in fragmented and unstable habitats (Suarez et al. 2022; Eriksson et al. 2014).

Dispersal is also central to several problems in contemporary biology. Greater insight into dispersal processes will hopefully enable us to better understand and predict species’ ability to cope with anthropogenic changes to climates and landscapes (Travis et al. 2013). This will also make us better prepared to combat the introduction and spread of non-native species, a major driver of biodiversity loss (Renault et al. 2018). Research into the factors underlying intraspecific variation in dispersal, and the traits associated with dispersal strategies will also allow the integration of evolutionary theory into pest management (Mazzi and Dorn 2012); improved knowledge of the movement of pests will allow us to better forecast outbreaks, design pest management strategies and improve global food security (Jeger 1999).

A genetic basis of dispersal-related traits has been shown in a range of species (Saastamoinen et al. 2018), though the mechanistic basis of effects on dispersal are more difficult to study and often remain unknown. The genetic architecture of dispersal is usually thought to be polygenic (e.g. Jordan et al. 2012); however, genes with large effects on dispersal have been identified across taxa; and can be broadly separated into those with either metabolic (Niitepõld and Saastamoinen 2017) or neurophysiological (Trefilov 2000; Fidler et al. 2007; Krackow and Konig 2008; Sokolowski 1980; Anreiter and Sokolowski 2019) effects on movement. Neurophysiological variation can underlie suites of traits within an individual, leading to consistent and correlated responses that differ among individuals, called personality, typically studied in terms of traits such as activity and boldness (Roche et al. 2016). Non-behavioral traits can also be part of syndromes, for example genetic differences in morphology can be correlated with, or directly affect, dispersal. Classically, a wing-polyphenism in the pea aphid *Acyrthosiphon pisum* is under the control of a single sex-linked locus determining the presence/absence of wings (Caillaud et al. 2002), however, in other species morphological differences associated with movement can be more quantitative. Within species, overall body size often covaries with dispersal, though the picture is complex and the direction of the relationship is dependent on the system (Bowler and Benton 2005). In many cases the key morphological trait may be the size or shape of a specific functional structure, such as a leg or pelvis (Losos 1990; Hudson et al. 2016).

The red flour beetle *Tribolium castaneum* (Coleoptera, Tenebrionidae) is a globally significant post-harvest agricultural pest and an established model organism (Boxall 2001; El-Aziz 2011; Pointer et al. 2021). Dispersal is an important aspect of *Tribolium* ecology (Dawson 1977) and dispersal within and between food storage facilities frustrates efforts to control their impact as pests (Semeao et al. 2013). *Tribolium castaneum* moves by both walking and flight, but flies under only certain environmental conditions (Drury et al. 2016), is only very rarely seen to fly inside and is caught outside in traps targeting walking individuals (Semeao et al. 2013). Therefore, while longer dispersals are likely by flight (Ridley et al. 2011), walking is the most common mode of dispersal. A body of previous work in this system has used artificial selection or experimental evolution to demonstrate strong genetic control of locomotive dispersal and rapidly generate large phenotypic differences in dispersal propensity between lines (E.g. Prus (1966); Ogden 1970a); Ritte and Lavie (1977); Korona (1991); Melbourne and Hastings (2009); Weiss-Lehman et al. (2017); Ochocki and Miller (2017); Ruckman and Blackmon 2020; Arnold et al. 2023; Pointer et al. 2023). Correlations between dispersal and other life history traits have been found (Ritte and Lavie 1978; Lavie 1981; Zirkle et al. 1988; Pointer et al. 2024), however, the traits providing the proximate mechanisms underlying differences in dispersal have received little investigation outside of morphology (Arnold et al. 2017, 2023). Even here, results are equivocal, with leg length seen to vary positively with movement ability (Arnold et al. 2017), negatively with dispersal (Arnold et al. 2023) and variously with walking distance (Matsumura and Miyatake 2018, 2019; Matsumura et al. 2019). Hence, it is not currently known how dispersal is evolving in these populations, or which traits are able to respond to novel rapid selection on dispersal. Further, lines subject to negative selection for dispersal in Pointer et al. (2023) had the greatest phenotypic response, showing almost no dispersal propensity after five generations of selection. Identifying traits responsible for a loss of dispersal may be of particular interest in fields where the spread of organisms may be problematic, such as invasion biology or pest management.

Here we use lines of *T. castaneum* previously selected for differential dispersal (Pointer et al. 2023) to investigate associations between dispersal and other traits to understand the proximate mechanism leading to differences in dispersal tendency, and determine whether dispersal in this system is part of a behavioral syndrome. Specifically we test for differences in activity and movement patterns, morphology, and use of the surface of the habitat medium, that could result in the observed differences in levels of dispersal.

## Methods

### Beetles and Dispersal Propensity

The *Tribolium castaneum* beetles used in this study were from 44 experimental lines: 16 high dispersal lines and 16 low dispersal lines (referred to collectively as dispersal regimes) and 12 unselected control lines from the same original Krakow super-strain (KSS) stock (Laskowski et al. 2015), maintained under the same conditions as the selection lines for 5 generations prior to experiments. Full details of the selection experiment and husbandry procedures can be found in Pointer et al. (2023) but in brief, high (1–16 H) and low (1–16 L) dispersal lines were generated by five generations of divergent artificial selection, using a dispersal assay. In this assay, each individual was given three opportunities to “disperse” from a mixed-sex group of 200 conspecifics (i.e., leave a patch of suitable habitat (120 × 120 × 200 mm container filled to 50 mm with a 9:1 mixture of organic wheat flour and brewer’s yeast, and topped with oats for traction); cross a short distance of unsuitable habitat (150 mm of plastic tubing) and not return – see Fig. S1). Individuals that dispersed three times out of the three opportunities were considered to display a dispersive phenotype, and individuals that dispersed zero times from the three opportunities were considered to display a not-dispersive phenotype. Individuals of each of these phenotypic extremes were bred to produce the subsequent generation, while intermediate phenotypes were discarded. After a single generation of selection, mean dispersal phenotypes (measured as the mean number of dispersal events per individual out of three opportunities) between the treatments were significantly different. After five generations of selection, dispersal phenotypes were strongly divergent, and the distributions of dispersal phenotypes between the two treatments were non-overlapping (Pointer et al. 2023). Thereafter, in order to reduce experimental effort, selection was applied only in even numbered generations up to generation 16. In generation 17 we conducted an assay - using the above procedure as above - on populations of 200 beetles randomly selected from each experimental line. Individuals were marked after each dispersal opportunity and dispersal propensity in each line was scored as the mean number of realised dispersals per individual (out of the three opportunities).

### Activity and Movement Pattern

For an individual to disperse in an assay, it had to encounter the opening of the tube to the second container of the dispersal arena (Fig. S1). More active individuals would be more likely to encounter the opening and therefore disperse more often than less active individuals. Thus it is possible that differential levels of locomotor activity and/or movement are driving differential dispersal in our selection lines, so we developed an activity assay to test for this difference.

Activity arenas were constructed by cutting around the sides of a 1.2 L plastic tub 20 mm from its bottom, to remove the base. This was then attached to a large white clay tile wrapped in laboratory tissue paper, using hot glue on the outer surface (Fig. S2A). This created a square area within which beetles were able to grip and could move freely, but could not escape by climbing the smooth walls of the tub. A set of twelve arenas were arranged such that each was positioned directly below a video camera (models: Sony HDRCX115E; Sony HDRCX190E; Sony HDRCX405) mounted to a horizontal board 300 mm above (Fig. S2B). Arenas were lit by LED strips attached to the underside of the board. Pupae from each of the 44 experimental lines were sexed and sorted to form test populations of 10 individuals at 1:1 sex ratio, as mating status has been shown to alter movement pattern (Wexler et al. 2017). At this stage the experiment was blinded, line identities were replaced with three figure codes to mitigate unconscious bias during data collection and analysis. Two temporal blocks were used, block 1 consisted of high dispersal lines 1–8, low dispersal lines 1–8 and control lines 1–6, block 2 consisted of high dispersal lines 9–16, low dispersal lines 9–16 and control lines 7–12. At 7 ± 1 days post-eclosion, each test population was placed into an experimental arena, given 10 min acclimation time, then recorded for 10 min at 25 frames per second. Assays were conducted either in the afternoon (1400–1600), or in the evening (1700–1900) in order to include the time of day when activity is known to peak in this species (Rafter et al. 2019). The timing of assays was randomised across all treatments. Event logging software BORIS (Friard and Gamba, 2016) was used to manually record the time of any escapes from activity arenas for each video.

### Surface Affinity

Over the course of the selection experiment, we observed anecdotally that fewer beetles were present on the fodder surface in replicates of low dispersal selection lines than high dispersal lines. It is possible that individual decisions on whether to remain on the fodder surface are driving differences in dispersal (as only individuals on the surface encounter the dispersal tube). To test this we assayed the surface affinity of populations under the same conditions experienced during dispersal assays. Arenas were identical to pot A of the dispersal arenas used during artificial selection for dispersal (Fig. S1), but lacking the opening of the tube leading to a second pot. Test populations were placed onto the surface of the fodder in an arena. After 2 h, photographs were taken of the surface of the fodder and the number of beetles remaining on the surface of the fodder in each replicate was determined. This may have been an underestimate as some individuals were likely obscured by the oats, but gave a minimum value.

To determine if surface affinity is under genetic control we conducted selection on this trait over a single generation. The experimental setup was exactly as described above, but in addition the arena contained a horizontal slot through which a thin plastic separator could be pulled, partitioning the arena contents (Fig. S2). The slot was positioned such that, when the arena contained 250 ml of fodder and the separator was pulled across, fodder within 8 mm of the fodder surface was above the partition, and fodder more than 8 mm from the fodder surface was below the partition. Early trials suggested that an 8 mm distance provided the best separation of surface from not-surface individuals. Populations of 200 unselected stock beetles were placed into arenas, and after two hours the separator was pulled across and the beetles in each partition sieved from their fodder. Thirty individuals from each group of “surface” and “not-surface” beetles were then isolated together for three days, before being transferred to fresh fodder to oviposit. The adults were removed after 7 days and the eggs left to develop to adulthood. At 12 ± 3 days post-eclosion the offspring were assayed using the same procedure (above) as their parents to ascertain their surface affinity. The timing of assays was controlled, as above, by randomising all replicate trials across periods before and during the peak of activity.

### Morphology

Beetles from the 44 experimental lines were collected 12 ± 3 days post eclosion and frozen at -80 C until thawed for dissection. The left elytrum and rear left leg were dissected out from 780 individual beetles, 15 of each sex from each of lines 1–10 L, 1–10 H and 1–6 C. Dissections were carried out under an Olympus SZX9 microscope using fine tip watchmaker’s forceps, in 30 µl insect saline solution on a clean glass slide. Body parts were imaged under 4X using a dark field phase contrast microscope and an Olympus BX41 camera through GX capture v8.5 software. A 1 mm calibration slide was used between sessions. ImageJ software (Schneider et al. 2012) was used to measure the length of the elytrum, femur, tibia and first tarsus segment, along with femur width at the widest point. *Tribolium* leg segments shown some curvature; however, we were interested in their linear extent as the determinant of stride-length. For individuals where an accurate measurement could not be made from the photograph, no measurement was taken. To standardise the procedure as far as possible, all dissection and photography was carried out by RV, using the outer surfaces of the right leg, and all measurements were taken by MDP. For a subset of 90 individuals measurements were repeated four days after the last initial measurements for analysis of repeatability.

### Statistical Methods

All data wrangling and analyses were performed in R (ver.4.3.1; R core team 2023). Mixed models were fitted using package ‘lme4’ (Bates et al. 2015) with p-values added with ‘lmerTest’ (Kuznetsova et al. 2017). Summary statistics are presented as means ± standard error throughout.

To test for a difference in dispersal propensity we fitted a linear model (Table 1). The response variable was a population-level measure of the mean number of dispersals per individual, this was used as the density dependence of dispersal behavior (e.g. Ogden 1970b) means that individual dispersal events are non-independent.

Movement tracking was performed on 110 10 min (15000 frame) video clips using a machine learning algorithm in the software package Loopy (http://loopbio.com/loopy/; settings are provided in Table S1). Additionally, eight 400 frame clips were human-annotated to ground-truth the model. While individuals were tracked, due to the difficulty associated with tracking individuals moving over each other, tracks were aggregated to give a measure of activity at the population-level for each replicate. Location data from tracking software was used to derive three metrics of activity for each population.

1) As a replicate-level measure of activity, path lengths were calculated from location data as the total distance traveled, in pixels, by each individual per second (details provided in Supplementary methods). Pearson’s correlation was very high between path distances calculated from human-annotated clips and the same clips tracked with the machine learning model providing confidence in the tracking model (*n* = 8, rho = 0.99, 95%CIs = 0.98, 0.99).

2) Sinuosity of beetle paths was calculated per replicate, as a measure of movement tortuosity, according to the method of (Benhamou 2004); details provided in Supplementary methods).

3) Edge affinity was computed for each replicate as the proportion of recorded beetle locations that were within 10 mm of an edge of the arena, with edges of the arena defined as the maximum and minimum X and Y locations recorded across the whole replicate recording.

During data exploration, all records from a single camera (11) in block 2 were identified as extreme outliers and omitted from the analysis. The same linear mixed effects model (GLMM) structure was used to model the three movement metrics, path length, sinuosity and edge affinity (Table 1). The sinuosity model was fitted twice, once for each of two rediscretisation distances (*p* = 10 and *p* = 20), a parameter used to control for non-independence of consecutive turns (see Supplementary methods), results presented are from models using data where *P* = 10, however using *P* = 20 did not qualitatively change the results and these are presented in Supplementary results.

The surface affinity of populations and the genetic control of surface affinity were each analyzed with GLMMs (Table 1). Repeatability of morphological data was assessed by calculating Spearman’s rank correlation between repeated measurement taken from the same individual. As all morphological variables were highly correlated (*r* > 0.6), we condensed the information using factor analysis, on a correlation matrix using the function *prcomp* from the R package ‘stats’ (R Core Team 2023). However, factor analysis cannot deal with missing data, of which there was a high proportion for femur length due to residual thorax tissue preventing accurate measurement; we therefore took forward only records for which all metrics had been quantified (n = 330). A single principal component (PC1) accounted for 74% of the total variation (Fig. S4). Each variable contributed roughly equally to PC1 (23–26%) and all were negatively correlated (Table S2), we therefore flipped the sign of PC1 to make the interpretation more intuitive. The remaining PCs each captured ≤ 10% of the total variation and were less biologically interpretable. We therefore took forward only PC1 into further analyses, as a proxy for overall body size. We used GLMMs to test separately for overall size differences (represented by PC1) between selection regimes and sexes (Table 1) and for a difference in leg length relative to overall body size (Table 1). Where interaction terms were non-significant they were removed and models refit to test the independent effects of fixed factors.

**Table 1:** Model structure used in each test of a specific movement variable

Test	Independent variable	Fixed factor/s	Random factor/s
Dispersal	Mean dispersals per individual	Dispersal regime	
Path length	Path length	Dispersal regime	Block ID, Line ID, camera ID
Sinuosity	Sinuosity	Dispersal regime	Line ID, camera ID
Edge affinity	Edge affinity	Dispersal regime	Line ID, camera ID
Surface affinity - populations	Number on surface	Dispersal regime	Block
Genetic control of surface affinity	Proportion on surface	Selection regime	Block
Overall body size	Morphological PC1	Dispersal regime*sex	Line ID
Relative leg length	Leg length (femur + tibia)	Dispersal regime *sex, morphological PC1	Line ID

Models containing only fixed factors were fitted as GLMs, those containing random factors were fitted as GLMMs. Interactions are shown with the ‘*’ symbol, representing an interaction term alongside each of the interacting terms added individually

## Results

### Dispersal Propensity

**Fig. 1:**
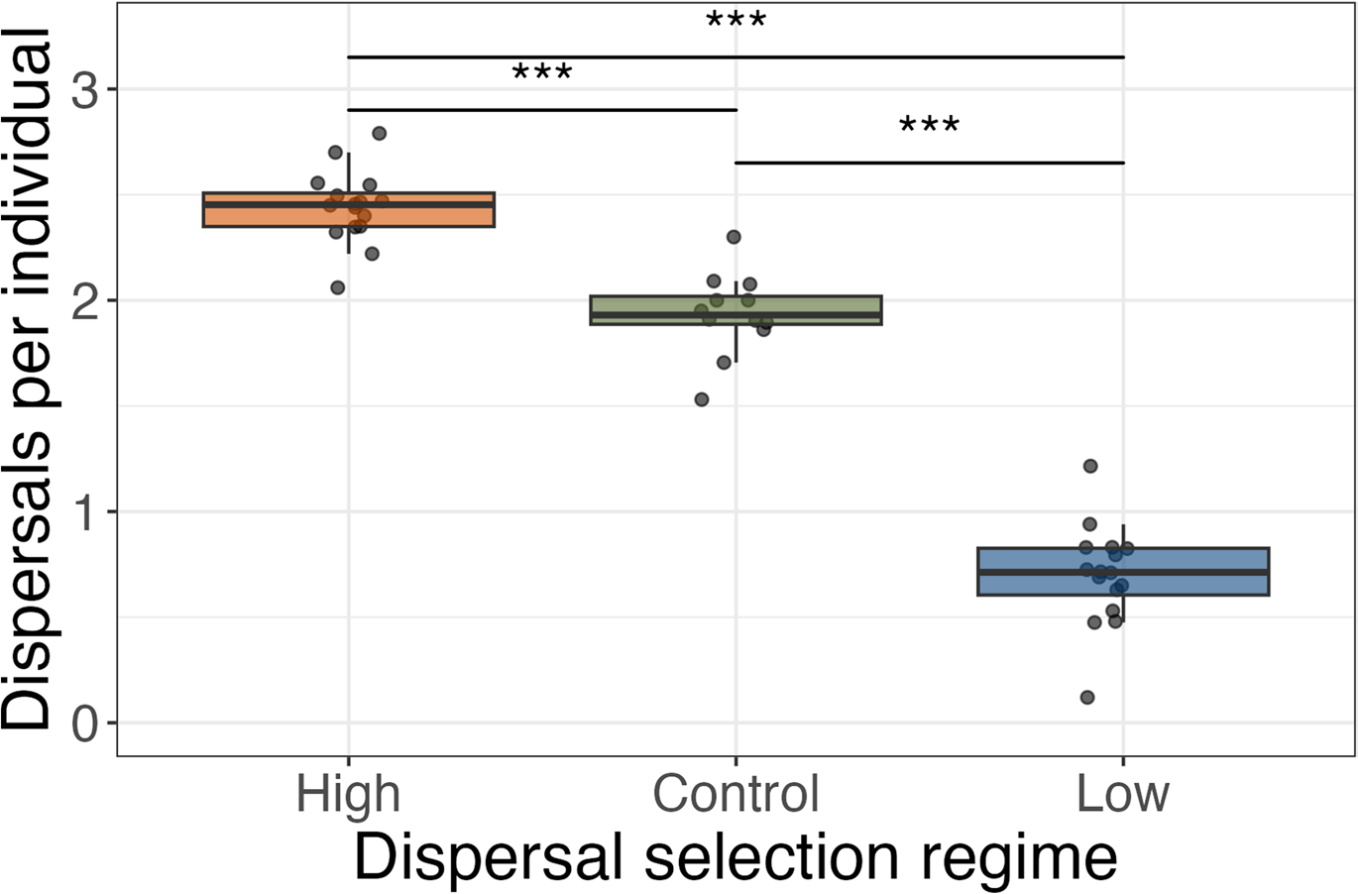
Mean number of dispersals per individual (out of a maximum of three) in populations of 200 *Tribolium castaneum* flour beetles, taken from lines artificially selected for high (*n* = 16) or low (*n* = 16) levels of dispersal behavior or from unselected control lines (*n* = 12)

Beetles from low dispersal lines (0.70 ± 0.06) dispersed significantly less than those from high dispersal lines (2.44 ± 0.04; LM, β=-1.74, se = 0.07, *p* < 0.001; Fig. 1) and significantly less than unselected control lines (1.94 ± 0.06; LM, β=-1.23, se = 0.08, *p* < 0.001; Fig. 1). High dispersal lines dispersed more than control lines, with the magnitude of the difference being greater than that between low dispersal lines and controls (LM; β = 0.51, se = 0.08, *p* < 0.001; Fig. 1).

### Activity and Movement Pattern

**Fig. 2:**
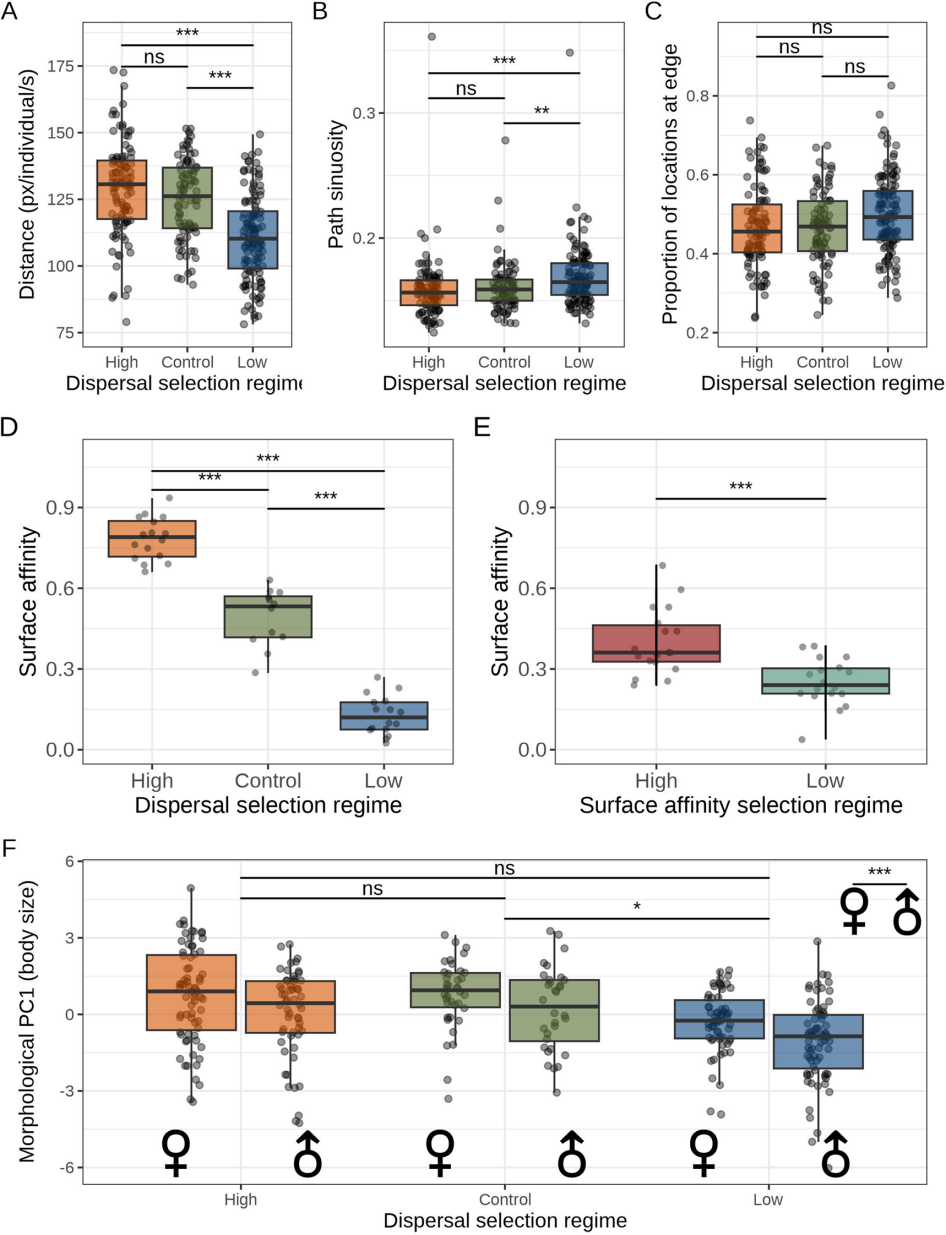
Variation in traits across selection lines. Activity and movement traits in dispersal selection lines are shown as (**A**) tracked path length; **B** Path sinuosity; **C** Edge use. Surface affinity (proportion of 200 beetles remaining on the surface after 2 h) in lines artificially selected for (**D**) dispersal propensity over nine generations and (**E**) surface affinity over a single generation. **F** Body size represented by morphological PC1 in dispersal selection lines. Boxes display interquartile ranges and bold lines show medians

Average distances (pixels second^−1^) traveled by beetles from low dispersal lines (111 ± 1.46) were shorter than those traveled by either high dispersal (129 ± 1.76; GLMM: β = -17.94, SE = 3.14, *p* < 0.0001; Fig. 2A) or control (125 ± 1.6; β=-14.24, SE = 3.35, *p* < 0.001) regimes, which did not differ from each other (β = 3.70, SE = 3.40, *p* = 0.28).

Beetles from low dispersal lines moved with more sinuous paths than did beetles from high dispersal lines (*P* = 10, GLMM: *P* = 10, β = 0.01, SE = 0.003, *p* < 0.001; Fig. 2B), and also moved more sinuously than control lines (*P* = 10, β = 0.009, SE = 0.003, *p* = 0.01). The control treatment sinuosity was intermediate between high and low lines, but did not differ from high lines (*P* = 10, β=-0.002, SE = 0.003, *p* = 0.48).

Edge use in low dispersal lines (0.50 ± 0.10) was not significantly higher than in high dispersal lines (0.47 ± 0.11; β = 0.03, SE = 0.02, *p* = 0.06; Fig. 2C) or controls (0.47 ± 0.09; β = 0.03, SE = 0.02, *p* = 0.15). Edge affinity of high dispersal lines did not differ significantly from that of controls (β=-0.006, SE = 0.02, *p* = 0.76).

### Surface Affinity

The surface affinity of high dispersal lines (157.00 ± 3.48) was greater than that of low dispersal lines (57.90 ± 6.60, GLMM; β = 131.25, SE = 5.96, *p* < 0.001; Fig. 2D) and controls (99.2 ± 9.33, GLMM; β = 58.63, SE = 6.43, *p* < 0.001). Low dispersal lines had lower surface affinity than did controls (β=-72.63, SE = 6.43, *p* < 0.001).

The offspring of unselected stock parents collected from the fodder surface were found on the surface significantly more than the offspring of parents collected from below the surface of the fodder (GLMM; β = 0.15, SE = 0.03, *P* < 0.001, Fig. 2E).

### Morphology

Measurements of elytra length, femur length, femur width and tibia length were all highly repeatable (Spearman’s rank correlation, *p* < 0.001; Table S2). Length of the first tarsus segment had substantially lower repeatability (*N* = 55, *r* = 0.73, *p* < 0.001) and was dropped from further analyses.

Sex was a strong predictor of PC1, with males being smaller than females (-0.42 ± 0.14 versus 0.39 ± 0.12 respectively; GLMM; β = 0.82, SE = 0.16, *p* < 0.001; Fig. 2F). PC1 was significantly lower in the low selection lines (-0.70 ± 0.13) than in controls (0.45 ± 0.16; GLMM, β=-1.10, SE = 0.52, *p* = 0.049), but did not differ either between high dispersal lines (0.52 ± 0.18) and controls (β=-0.32, SE = 05.2, *p* = 0.55), or between low and high dispersal lines (GLMM; β=-0.79, SE = 0.4, *p* = 0.09). There was no significant interaction effect between selection regime and sex on PC1 (GLMM; all *p* > 0.07). When controlling for PC1 as a measure of body size, leg length did not differ significantly between any combination of selection regime and control lines (GLMM; all *p* > 0.42), nor between males and females (*p* = 0.07), nor was any interaction significant between the effects of selection regime and sex (GLMM; all *p* > 0.68).

## Discussion

We found that *Tribolium castaneum* populations selected for divergent dispersal behavior differ in levels of activity and movement pattern, use of the substrate surface and body size. The consistency of these effects across many independently evolving replicate lines (see Fig. 2) indicates that the coevolution of these traits is robustly repeatable. The tested traits are commonly seen as part of dispersal syndromes in other taxa, suggesting that dispersal in *Tribolium* should be considered in the context of a broad life-history strategy.

We observed that artificial selection on dispersal propensity generated significantly different levels of path sinuosity and activity in lines of *T. castaneum*. Previous studies in *Tribolium* have shown that movement distance is heritable, generating large differences in the trait under divergent artificial selection (Matsumura and Miyatake 2015), but have not assayed the dispersal propensity in these populations. Our finding agrees with many other studies across animals showing that dispersal is associated with exploratory activity (Krackow 2003; Cote et al. 2010b). The picture is similar for sinuosity, with exploratory individuals in many species moving on straighter paths (Brown et al. 2014; Klarevas-Irby et al. 2021), as we have shown for *T. castaneum*. Further, such activity and movement traits are commonly seen as personality traits and observed as components of behavioral syndromes, suites of traits that covary and show consistency within individuals (Wolf and Weissing 2012). In addition, we observe large variation within treatments of all activity and movement traits. High variance in movement traits, including dispersal itself, has also been seen as a feature of populations where dispersal is evolving (Melbourne and Hastings 2009; Ochocki and Miller 2017; Weiss-Lehman et al. 2017). However, we do not see similarly high variance in our measure of dispersal. Therefore, it may be that each movement trait is contributing only a small amount to the large overall difference in realised dispersal. It is also possible that the population-level approach we took to measuring activity and the AI tracking methodology introduced measurement error and inflated variances. If true, this may have prevented us from detecting differences in movement traits between high dispersal lines and controls, where the magnitude of the dispersal differences is less than between low dispersal lines and controls (reasons why dispersal may have responded more strongly to negative selection are discussed in more detail in Pointer et al. 2023).

For organisms living within a three-dimensional matrix - such as dead wood and grain masses, the ancestral and contemporary habitats of *T. castaneum* (Dawson 1977) - presence at the fodder surface increases risk. Therefore, an individual’s willingness to expose itself to this risk may well be indicative of boldness. Boldness - broadly defined as risk-taking (Sloan Wilson et al. 1994) - is another prominent animal personality trait, which shows considerable individual variation in a broad range of taxa, from humans to cockroaches (Sloan Wilson et al. 1994; Stanley et al. 2017). Our results show that individuals from high dispersal lines spend more time at the fodder surface than those from low dispersal lines, suggesting a higher level of boldness. Dispersal is known to correlate with boldness across animal taxa, with important implications for invasiveness (Cote et al. 2010a; Myles-Gonzalez et al. 2015) and are thought to be mediated through common physiology and/or endocrinology (Cote et al. 2010a). This study examined surface use, as a measure of boldness, in the absence of predators. As predation represents one of the main risks to individuals, it may be that the presence of predators would modify surface use, and thus change, or reveal more about variation in, individual boldness. In the cockroach *Gromphadorhina portentosa*, past predator interaction reduces boldness (but not activity; McDermott et al. 2014). In contrast, boldness and predator avoidance represent separate axes of behavior in the ground beetle *Nebria brevicollis* (Labaude et al. 2018). Measuring surface use in the presence versus absence of a predator would resolve which of these patterns is followed in *Tribolium*, and even whether the response to predation risk varies across dispersal phenotypes. Additionally, time of day might play a role in mediating boldness and movement traits. Our measures here were averages across the on- and off-peak activity periods for *Tribolium* (Rafter et al. 2019), but future work performing separate tests across these times would reveal if dispersers show differential circadian cycles of activity than not-dispersers, however this was beyond the scope of the current project.

In addition to the effects of behavior, animal movement patterns also often differ as a result of quantitative individual differences in morphology. For example, sprint speed in lizards is dependent upon leg length (Losos 1990). Current evidence for associations between morphology and movement in *Tribolium* is not simple to interpret. Greater leg length, but not body size, was related to increased movement ability when tested within a single generation (Arnold et al. 2017), suggesting a simple mechanistic relationship between morphology and movement ability. Recent work measuring body size and femur length following artificial selection on dispersal showed that body size and mass were inversely related to dispersal propensity (Arnold et al. 2023). In addition, both body size and leg length (controlling for body size) were correlated with walking distance in lines selected for death-feigning duration (Matsumura and Miyatake 2019). However, separate studies have shown the opposite relationship, with shorter leg length in males artificially selected for walking distance (Matsumura et al. 2019), and no relationship in females (Matsumura and Miyatake 2018). In the current study we did not test morphology directly against movement, but tested both in lines selected for differential dispersal propensity. We found that lines selected for low dispersal propensity have experienced a concomitant reduction in body size - so in that sense dispersers have longer legs - but that leg length did not differ relative to body size. Hence, our results agree with the general expectation that dispersive individuals are larger (Renault 2020), and with specific examples where invasive beetles are larger at the range front (Laparie et al. 2013; Yarwood et al. 2021). However, we find a contrast with Arnold et al. (2023), which at first appears to be very similar work to ours in terms of comparing morphology in dispersal lines. On closer examination, the dispersal assays employed in each study differ markedly, with the present study requiring walking, whereas Arnold et al. required individuals to climb to disperse. As Arnold et al. note, “smaller individuals are biomechanically and energetically better suited to dispersal by climbing”, in contrast to dispersal by walking, which may explain the differential findings. Interestingly, Arnold et al. (2023) also find that the sexes respond differently to selection on dispersal when using a design that selects separately on males and females in isolation. Thus, in their study dispersal included mate-finding behavior. In contrast, we aimed to exclude mate-finding by selecting on dispersal in mixed-sex groups of beetles and found no difference in morphological response between males and females. In this light, our results support the suggestion of Arnold et al. that mate-finding, which drives higher dispersal in males in the absence of females (Prus 1966; Ogden 1970b), may determine sex differences in morphology. Overall, such patterns serve to highlight the complexity of studying dispersal, and support the thesis that measures of movement traits are sensitive to differences in experimental design, setup and arena settings, as found by a recent study using *Tribolium* (Scharf et al. 2023).

Body size is a trait commonly seen as part of dispersal syndromes - the suites of traits associated with dispersal - though the direction of the relationship between size and dispersal is dependent on the specifics of the system, and even on environmental conditions (Bowler and Benton 2005). Dispersal in *Tribolium* is usually thought of as a way for small individuals who may be poor competitors to avoid resource competition (Zirkle et al. 1988; Arnaud et al. 2005), so a larger body size of dispersers suggests that other factors might also be at work. Greater size of dispersers is observed in many animal species, including insects (Anholt 1990; Bowler and Benton 2005). Possibly larger size is mechanistically beneficial for efficient movement, or it may be that larger size/better condition is required to undertake movement (Bowler and Benton 2005).

Since dispersal is “any movement of individuals or propagules with potential consequences for gene flow across space” (Ronce 2007), rather than a behavior per se, individual activity can be defined as a tendency that contributes to dispersal behavior (Benton et al. 2012). High dispersal lines were more active and moved on straighter paths, and it is difficult to imagine that such behavior would not tend to be more dispersive than the converse. Moving with greater activity and in straighter lines would achieve a given dispersal distance in less time, with less energy expenditure and less exposure to risk. Surface use also seems straightforwardly related to dispersal; the first stage of dispersal is emigration; in order to disperse an individual must leave its initial location (Clobert et al. 2012), a necessary first step in dispersing to a new patch might be choosing not to burrow into the substrate but to remain on the surface. We also observed that surface use responded significantly to a single generation of selection, suggesting that this trait has a simple genetic basis. There is evidence for an oligogenic genetic architecture of dispersal in *Tribolium* (Pointer et al. 2023), and the tight correlation between dispersal and surface use may point to either a shared mechanism or causal relationship between these traits. As commonly associated personality-related traits, activity and boldness may be underpinned by shared neurophysiological pathways, such as those related to dopamine signalling (Silva et al. 2020; Wu and Seebacher 2022). Loci with neurophysiological effects on dispersal include those relating to dopamine signalling in birds and mammals (Fidler et al. 2007; Krackow and Konig 2008; Trefilov 2000). In insects, the *foraging* gene, coding for a signalling regulator, affects dispersal in the diptera, lepidoptera and orthoptera (Sokolowski 1980; Anreiter and Sokolowski 2019). Dopamine and other biogenic amines have been previously linked to behavior in *T. castaneum* (Miyatake et al. 2008; Nishi et al. 2010). It would be instructive to investigate molecular genomic variation between the lines used in this study and sites related to dopamine signalling seem to be reasonable a priori candidates based on our results.

Overall, our results suggest that dispersal is associated with a suite of traits in *T. castaneum*. The presence of a such a dispersal syndrome, or personality-dependent dispersal, is known from many taxa, from single-celled organisms to mammals, including many insects (Cote et al. 2010a; Clobert et al. 2012; Fronhofer et al. 2018; Renault 2020). Further, dispersal syndromes may be part of overarching pace-of-life syndromes linking personality to multiple behaviors (Réale et al. 2010). Some traits in a syndrome may directly assist dispersal, whereas others mitigate costs, although the line between these categories is often blurred (Cote et al. 2010a). The set of traits tested in this study: activity pattern; morphology; surface use, all covary with dispersal tendency in a direction that plausibly enhances the dispersal in high dispersal lines and/or mitigates the costs of dispersing. Deeper understanding of the dispersal syndrome in *T. castaneum* requires genomic study and multivariate analysis of a broad range traits, across a large set of lines. Ideally these lines would be isogenic to remove individual-level variation and enable characterisation of traits and genotypes in genetically identical, and genetically stable, populations across space and time. Dispersal interacts with many aspects of ecology and life-history, and traits of interest might include those related to development, reproduction, population dynamics and social environment, physiology, and senescence – all of which have been subjects of *Tribolium* research, but not in a framework capable of resolving their interrelatedness or genetic underpinnings. Knowledge of eco-evolutionary dynamics is key to understanding biogeography and changes in range (Wellenreuther et al. 2022), which is especially important for species with significant effects on the environment or human populations. Our findings suggest that suites of correlated traits enable species to respond to selection on dispersal; therefore, this context must be considered when investigating the limits of dispersal evolution, and in attempting to predict and control the spread of organisms such as invasive species, crops pests and disease vectors.

## Supplementary Information

Below is the link to the electronic supplementary material.

ESM 1 (DOCX 923 KB)

## Data Availability

The data files and analysis scripts used in this study are openly available from Mendeley data (10.17632/zcb97xf8xt.1).

## References

[CR1] Anholt BR (1990) Size-biased dispersal prior to breeding in a damselfly. Oecologia 83:385–387 28313011 10.1007/BF00317564

[CR2] Anreiter I, Sokolowski MB (2019) The foraging gene and its behavioral effects: pleiotropy and plasticity. Annu Rev Genet 53:373–392 31487469 10.1146/annurev-genet-112618-043536

[CR3] Arnaud L, Brostaux Y, Lallemand S, Haubruge E (2005) Reproductive strategies of Tribolium flour beetles. J Insect Sci 5:33 17119615 10.1093/jis/5.1.33 PMC1615240

[CR4] Arnold PA, Cassey P, White CR (2017) Functional traits in red flour beetles: the dispersal phenotype is associated with leg length but not body size nor metabolic rate. Funct Ecol 31:653–661

[CR5] Arnold PA, Cassey P, White CR (2023) Morphological shifts in response to spatial sorting of dispersal behavior in red flour beetles across multiple generations. J Zool 320:131–142

[CR6] Bates D, Mächler M, Bolker B, Walker S (2015) Fitting linear mixed-effects models using lme4. J Stat Softw 67:1–48

[CR7] Benhamou S (2004) How to reliably estimate the tortuosity of an animal’s path: straightness, sinuosity, or fractal dimension? J Theor Biol 229:209–220 15207476 10.1016/j.jtbi.2004.03.016

[CR8] Benton TG, Bowler DE, Clobert J (2012) Linking dispersal to spatial dynamics. In: Clobert J, Baguette M, Benton TG, Bullock JM (eds) Dispersal ecology and evolution. OUP, Oxford, pp 251–265

[CR9] Bowler DE, Benton TG (2005) Causes and consequences of animal dispersal strategies: relating individual behavior to spatial dynamics. Biol Rev Camb Philos Soc 80:205–225 15921049 10.1017/s1464793104006645

[CR10] Boxall RA (2001) Post-harvest losses to insects - a world overview. Int Biodeterior Biodegradation 48:137–152

[CR11] Brown GP, Phillips BL, Shine R (2014) The straight and narrow path: the evolution of straight-line dispersal at a cane toad invasion front. Proc Biol Sci 281:20141385 25297862 10.1098/rspb.2014.1385 PMC4213614

[CR12] Caillaud MC, Boutin M, Braendle C, Simon J-C (2002) A sex-linked locus controls wing polymorphism in males of the pea aphid, Acyrthosiphon pisum (Harris). Heredity 89:346–352 12399992 10.1038/sj.hdy.6800146

[CR13] Clobert J, Baguette M, Benton TG, Bullock JM (2012) Dispersal ecology and evolution. Oxford University Press

[CR14] Cote J, Clobert J, Brodin T, Fogarty S et al (2010a) Personality-dependent dispersal: characterization, ontogeny and consequences for spatially structured populations. Philos Trans R Soc Lond B 365:4065–4076 21078658 10.1098/rstb.2010.0176 PMC2992741

[CR15] Cote J, Fogarty S, Weinersmith K, Brodin T (2010b) and others Personality traits and dispersal tendency in the invasive mosquitofish (Gambusia affinis). Proc Biol Sci 277:1571–1579 20071380 10.1098/rspb.2009.2128 PMC2871838

[CR16] Dawson PS (1977) Life history strategy and evolutionary history of Tribolium flour beetles. Evolution 31:226–229 28567733 10.1111/j.1558-5646.1977.tb01001.x

[CR17] Drury DW, Whitesell ME, Wade MJ (2016) The effects of temperature, relative humidity, light, and resource quality on flight initiation in the red flour beetle, Tribolium castaneum. Entomol Exp Appl 158(3):269–74 27087697 10.1111/eea.12401 PMC4831652

[CR18] El-Aziz SEA (2011) Control strategies of stored product pests. J Entomol 8:101–122

[CR19] Eriksson A, Elías-Wolff F, Mehlig B, Manica A (2014) The emergence of the rescue effect from explicit within-and between-patch dynamics in a metapopulation. Proc R Soc London, SerB 281(1780):20133127 10.1098/rspb.2013.3127 PMC4027396 24523274

[CR20] Fidler AE, van Oers K, Drent PJ, Kuhn S, Mueller JC, Kempenaers B (2007) Drd4 gene polymorphisms are associated with personality variation in a passerine bird. Proc R Soc London, Ser B 274(1619):1685–91 10.1098/rspb.2007.0337 PMC1914334 17472912

[CR21] Friard O, Gamba M (2016) BORIS: a free, versatile open-source event-logging software for video/audio coding and live observations. MEE 7(11):1325–30

[CR22] Fronhofer EA, Legrand D, Altermatt F, Ansart A et al (2018) D bottom-up and top-down control of dispersal across major organismal groups. Nat Ecol Evol 2:1859–1863 10.1038/s41559-018-0686-0 30397298

[CR23] Hudson CM, McCurry MR, Lundgren P, McHenry CR et al (2016) Constructing an invasion machine: the rapid evolution of a dispersal-enhancing phenotype during the cane toad invasion of Australia. PLoS One 11:e0156950 10.1371/journal.pone.0156950 PMC5033235 27658247

[CR24] Jeger MJ (1999) Improved understanding of dispersal in crop pest and disease management: current status and future directions. Agric Meteorol 97:331–349

[CR25] Jordan KW, Craver KL, Magwire MM, Cubilla CE et al (2012) Genome-wide association for sensitivity to chronic oxidative stress in Drosophila melanogaster. PLoS One 7:e38722 10.1371/journal.pone.0038722 PMC3371005 22715409

[CR26] Klarevas-Irby JA, Wikelski M, Farine DR (2021) Efficient movement strategies mitigate the energetic cost of dispersal. Ecol Lett 24:1432–1442 33977638 10.1111/ele.13763

[CR27] Kokko H, López-Sepulcre A (2006) From individual dispersal to species ranges: perspectives for a changing world. Science 313(5788):789–91 16902127 10.1126/science.1128566

[CR28] Korona R (1991) Genetic basis of behavioral strategies. Dispersal of female flour beetles, Tribolium confusum, in a laboratory system. Oikos 62:265–270

[CR29] Krackow S (2003) Motivational and heritable determinants of dispersal latency in wild male house mice (mus musculus musculus). Ethology 109:671–689

[CR30] Krackow S, König B (2008) Microsatellite length polymorphisms associated with dispersal-related agonistic onset in male wild house mice (Mus musculus domesticus). Behav Ecol Sociobiol 62:813–820

[CR31] Kuznetsova A, Brockhoff PB, Christensen RHB (2017) lmerTest package: tests in linear mixed effects models. J Stat Softw 82:1–26

[CR32] Labaude S, O’Donnell N, Griffin CT (2018) Description of a personality syndrome in a common and invasive ground beetle (Coleoptera: Carabidae). Sci Rep 8:17479 30504923 10.1038/s41598-018-35569-z PMC6269510

[CR33] Laparie M, Renault D, Lebouvier M, Delattre T (2013) Is dispersal promoted at the invasion front? Morphological analysis of a ground beetle invading the Kerguelen Islands, merizodus soledadinus (Coleoptera, Carabidae). Biol Invasions 15:1641–1648

[CR34] Laskowski R, Radwan J, Kuduk K, Mendrok M et al (2015) Population growth rate and genetic variability of small and large populations of Red flour beetle (Tribolium castaneum) following multigenerational exposure to copper. Ecotoxicology 24:1162–1170 25920509 10.1007/s10646-015-1463-3

[CR35] Lavie B (1981) Longevity in lines of Tribolium castaneum selected for high and for low dispersal. J Gerontol 36:546–549 7264237 10.1093/geronj/36.5.546

[CR36] Lavie B, Ritte U (1978) The relation between dispersal behaviour and reproductive fitness in the flour beetle Tribolium castaneum. Can J Genet Cytol 20:589–595

[CR37] Losos JB (1990) The evolution of form and function: morphology and locomotor performance in West Indian anolis lizards. Evolution 44:1189–1203 28563896 10.1111/j.1558-5646.1990.tb05225.x

[CR38] Matsumura K, Miyatake T (2015) Differences in attack avoidance and mating success between strains artificially selected for dispersal distance in Tribolium castaneum. PLoS ONE 10:e0127042 10.1371/journal.pone.0127042 PMC4430303 25970585

[CR39] Matsumura K, Miyatake T (2018) Costs of walking: differences in egg size and starvation resistance of females between strains of the red flour beetle (Tribolium castaneum) artificially selected for walking ability. J Evol Biol 31:1632–1637 30055064 10.1111/jeb.13356

[CR40] Matsumura K, Miyatake T (2019) Lines selected for different durations of tonic immobility have different leg lengths in the red flour beetle Tribolium castaneum. Behavior 157:17–31

[CR41] Matsumura K, Archer CR, Hosken DJ, Miyatake T (2019) Artificial selection on walking distance suggests a mobility-sperm competitiveness trade-off. Behav Ecol 30:1522–1529

[CR42] Mazzi D, Dorn S (2012) Movement of insect pests in agricultural landscapes. Ann Appl Biol 160:97–113

[CR43] McDermott DR, Chips MJ, McGuirk M, Armagost F et al (2014) Boldness is influenced by sublethal interactions with predators and is associated with successful harem infiltration in Madagascar hissing cockroaches. Behav Ecol Sociobiol 68:425–435

[CR44] Melbourne BA, Hastings A (2009) Highly variable spread rates in replicated biological invasions: fundamental limits to predictability. Science 325:1536–1539 19762641 10.1126/science.1176138

[CR45] Miyatake T, Tabuchi K, Sasaki K, Okada K et al (2008) Pleiotropic antipredator strategies, fleeing and feigning death, correlated with dopamine levels in Tribolium castaneum. Anim Behav 75:113–121

[CR46] Myles-Gonzalez E, Burness G, Yavno S, Rooke A et al (2015) To boldly go where no goby has gone before: boldness, dispersal tendency, and metabolism at the invasion front. Behav Ecol 26:1083–1090

[CR47] Niitepõld K, Saastamoinen M (2017) A candidate gene in an ecological model species: phosphoglucose isomerase (pgi) in the glanville fritillary butterfly (Melitaea Cinxia). Ann Zool Fenn 54:259–273

[CR48] Nishi Y, Sasaki K, Miyatake T (2010) Biogenic amines, caffeine and tonic immobility in Tribolium castaneum. J Insect Physiol 56:622–628 20079743 10.1016/j.jinsphys.2010.01.002

[CR49] Ochocki BM, Miller TEX (2017) Rapid evolution of dispersal ability makes biological invasions faster and more variable. Nat Commun 8:14315 28128215 10.1038/ncomms14315 PMC5290149

[CR50] Ogden JC (1970a) Artificial selection for dispersal in flour beetles (Tenebrionidae: Tribolium). Ecology 51:130–133

[CR51] Ogden JC (1970b) Aspects of dispersal in Tribolium flour beetles. Physiol Zool 43:124–131

[CR52] Pointer MD, Gage MJG, Spurgin LG (2021) Tribolium beetles as a model system in evolution and ecology. Heredity 126:869–883 33767370 10.1038/s41437-021-00420-1 PMC8178323

[CR53] Pointer MD, Spurgin LG, Gage MJG, McMullan M, Richardson DS (2023) Genetic architecture of dispersal behavior in the post-harvest pest and model organism Tribolium castaneum. Heredity 131:253–262 37516814 10.1038/s41437-023-00641-6 PMC10539327

[CR54] Pointer MD, Spurgin LG, McMullan M, Butler S, Richardson DS (2024) Life history correlations and trade-offs resulting from selection for dispersal in Tribolium castaneum. J Evol Biol 37(7):48–757 10.1093/jeb/voae041 38654518

[CR55] Prus T (1966) Emigrational ability and surface numbers of adult beetles in 12 strains of Tribolium confusum (Duval) and T. Castaneum (Herbst). Ekol Pol Ser A 14:547–588

[CR56] R Core Team (2023) R: a language and environment for statistical computing. R Foundation for Statistical Computing, Vienna, Austria. https://www.R-project.org/. Accessed 20 June 2023

[CR57] Rafter MA, Muralitharan V, Chandrasekaran S, Mohankumar S et al (2019) Behavior in the presence of resource excess - flight of Tribolium castaneum around heavily-infested grain storage facilities. J Pest Sci 92:1227–1238

[CR58] Réale D, Garant D, Humphries MM, Bergeron P et al (2010) Personality and the emergence of the pace-of-life syndrome concept at the population level. Philos Trans R Soc Lond B 365:4051–4063 10.1098/rstb.2010.0208 PMC2992747 21078657

[CR59] Renault D (2020) A review of the phenotypic traits associated with insect dispersal polymorphism, and experimental designs for sorting out resident and disperser phenotypes. Insects 11:214 32235446 10.3390/insects11040214 PMC7240479

[CR60] Renault D, Laparie M, McCauley SJ, Bonte D (2018) Environmental adaptations, ecological filtering, and dispersal central to insect invasions. Annu Rev Entomol 63:345–368 29029589 10.1146/annurev-ento-020117-043315

[CR61] Ridley AW, Hereward JP, Daglish GJ, Raghu S et al (2011) The spatiotemporal dynamics of Tribolium castaneum (Herbst): adult flight and gene flow. Mol Ecol 20:1635–1646 21375637 10.1111/j.1365-294X.2011.05049.x

[CR62] Ritte U, Lavie B (1977) The genetic basis of dispersal behaviour in the flour beetle Tribolium. Can J Genet Cytol 19:717–722

[CR63] Roche DG, Careau V, Binning SA (2016) Demystifying animal ‘personality’ (or not): why individual variation matters to experimental biologists. J Exp Biol 219:3832–3843 27852750 10.1242/jeb.146712

[CR64] Ronce O (2007) How does it feel to be like a rolling stone? Ten questions about dispersal evolution. Annu Rev Ecol Evol Syst 38:231–253

[CR65] Ruckman SN, Blackmon H (2020) The march of the beetles: epistatic components dominate divergence in dispersal tendency in Tribolium castaneum. J Hered 111:498–505 32798223 10.1093/jhered/esaa030 PMC7525825

[CR66] Saastamoinen M, Bocedi G, Cote J, Legrand D et al (2018) Genetics of dispersal. Biol Rev Camb Philos Soc 93:574–599 10.1111/brv.12356 PMC5811798 28776950

[CR67] Scharf I, Hanna K, Gottlieb D (2023) Experimental arena settings might lead to misinterpretation of movement properties. Insect Sci 31:271–284 37231528 10.1111/1744-7917.13213

[CR68] Schneider CA, Rasband WS, Eliceiri KW (2012) NIH Image to ImageJ: 25 years of image analysis. Nat Methods 9:671–675 22930834 10.1038/nmeth.2089 PMC5554542

[CR69] Semeao AA, Campbell JF, Whitworth RJ, Sloderbeck PE (2013) Movement of Tribolium castaneum within a flour mill. J Stored Prod Res 54:17–22

[CR70] Silva PA, Trigo S, Marques CI, Cardoso GC et al (2020) Experimental evidence for a role of dopamine in avian personality traits. J Exp Biol 223:216449 10.1242/jeb.216499 31953366

[CR71] Sloan Wilson D, Clark AB, Coleman K, Dearstyne T (1994) Shyness and boldness in humans and other animals. Trends Ecol Evol 9:442–446 21236920 10.1016/0169-5347(94)90134-1

[CR72] Sokolowski MB (1980) Foraging strategies of Drosophila melanogaster : a chromosomal analysis. Behav Genet 10:291–302 6783027 10.1007/BF01067774

[CR73] Stanley CR, Mettke-Hofmann C, Preziosi RF (2017) Personality in the cockroach Diploptera punctata : evidence for stability across developmental stages despite age effects on boldness. PLoS ONE 12:e0176564 10.1371/journal.pone.0176564 PMC5425029 28489864

[CR74] Suárez D, Arribas P, Jiménez-García E, Emerson BC (2022) Dispersal ability and its consequences for population genetic differentiation and diversification. Proc R Soc London, Ser B 289(1975):20220489 10.1098/rspb.2022.0489 PMC9115014 35582805

[CR75] Travis JMJ, Delgado M, Bocedi G, Baguette M et al (2013) Dispersal and species’ responses to climate change. Oikos 122:1532–1540

[CR76] Trefilov A, Berard J, Krawczak M, Schmidtke J (2000) Natal dispersal in rhesus macaques is related to serotonin transporter gene promoter variation. Behav Genet 30:295–301 11206084 10.1023/a:1026597300525

[CR77] Weiss-Lehman C, Hufbauer RA, Melbourne BA (2017) Rapid trait evolution drives increased speed and variance in experimental range expansions. Nat Commun 8(1):14303 28128350 10.1038/ncomms14303 PMC5290145

[CR78] Wellenreuther M, Dudaniec RY, Neu A, Lessard J-P et al (2022) The importance of eco-evolutionary dynamics for predicting and managing insect range shifts. Curr Opin Insect Sci 52:100939 10.1016/j.cois.2022.100939 35644339

[CR79] Wexler Y, Wertheimer K-O, Subach A, Pruitt JN et al (2017) Mating alters the link between movement activity and pattern in the red flour beetle: the effects of mating on behavior. Physiol Entomol 42:299–306

[CR80] Wolf M, Weissing FJ (2012) Animal personalities: consequences for ecology and evolution. Trends Ecol Evol 27:452–461 22727728 10.1016/j.tree.2012.05.001

[CR81] Wu NC, Seebacher F (2022) Physiology can predict animal activity, exploration, and dispersal. Commun Biol 5:109 35115649 10.1038/s42003-022-03055-y PMC8814174

[CR82] Yarwood E, Drees C, Niven JE, Gawel M et al (2021) Sex differences in morphology across an expanding range edge in the flightless ground beetle, Carabus hortensis. Ecol Evol 11:9949–9957 34367551 10.1002/ece3.7593 PMC8328432

[CR83] Zirkle DF, Dawson PS, Lavie B (1988) An experimental analysis of the genetic relationships among life-history traits and emigration behavior in Tribolium Castaneum. Oikos 53:391–397

