# Supplementary material for: Traits Underlying Experimentally Evolved Dispersal Behavior in Tribolium castaneum
Source: J Insect Behav. 2024 Sep 27;37(3-4):220–32. doi: 10.1007/s10905-024-09862-x (PMC11564205; doi:10.1007/s10905-024-09862-x)
Supplement: Supplementary file 1 — (DOCX 923 KB) [file 10905_2024_9862_MOESM1_ESM.docx]

**Supplementary methods**

**
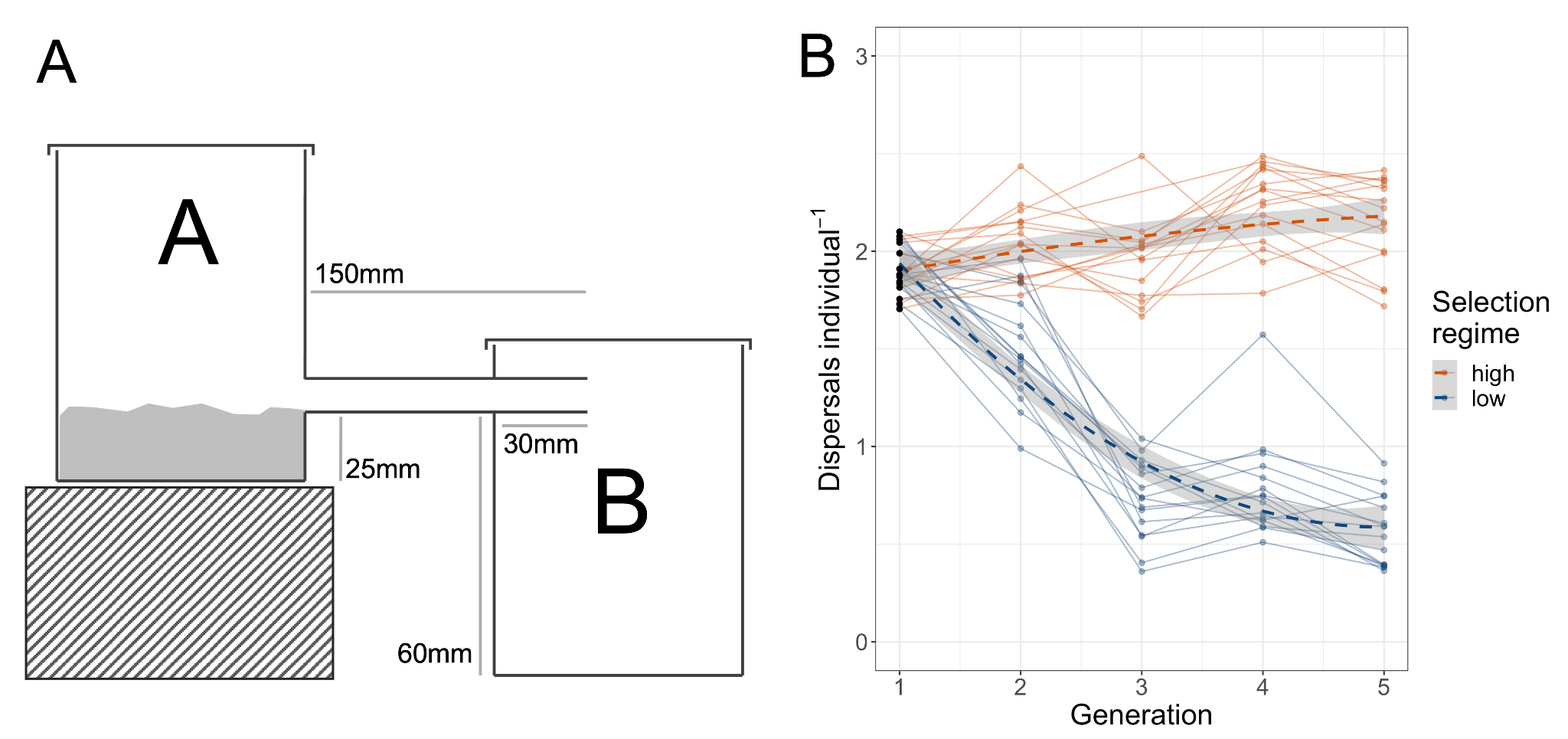
**

**Figure S1.** Experimental arena setup used to assay the dispersal behaviour of experimental *Tribolium castaneum* populations and provide a basis on which to artificially select individuals displaying high and low dispersal propensity.


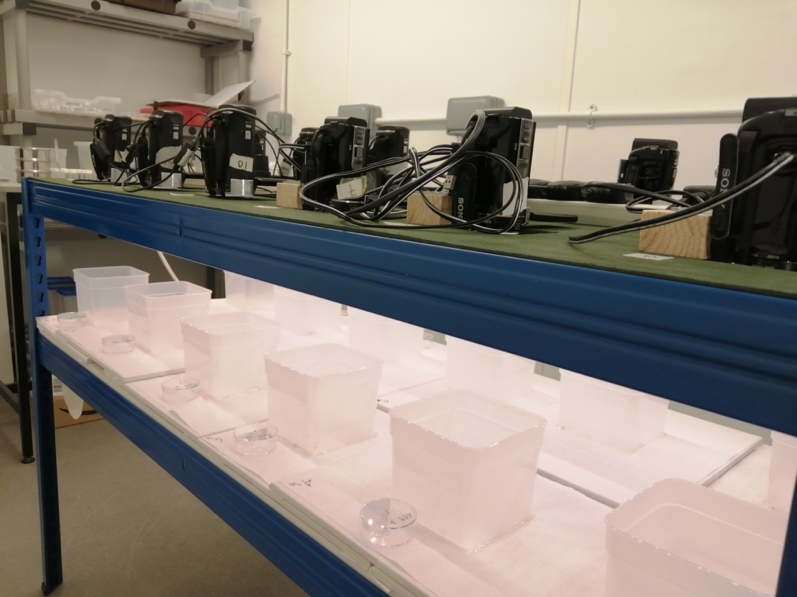


B


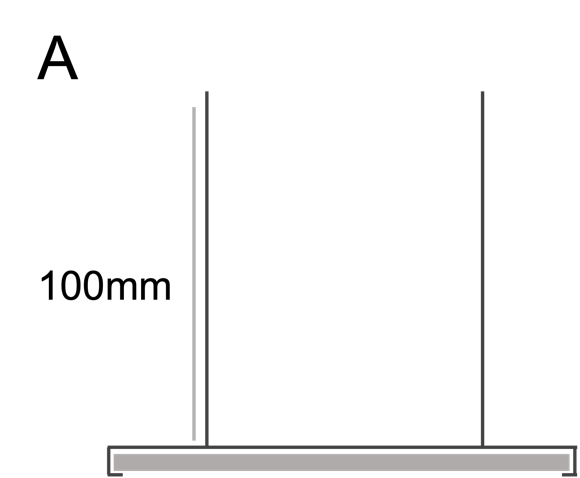


A

**Figure S2.** Experimental setup used to assay the movement behaviour of experimental *Tribolium castaneum* populations. A) Schematic of the arena, consisting of a plastic food container with the base removed, attached using hot glue to a ceramic tile wrapped in white laboratory tissue paper (shown in grey). B) How the arenas shown in A were arranged beneath a custom-made tabletop, to which 12 video cameras were mounted to film video clips.

**Activity and movement pattern**

Path lengths were calculated from location data for each detected object as:

$$\sum_{i=min}^{max} \sqrt{{(x_{i-1}-x_{i})}^{2}+{(y_{i-1}-y_{i})}^{2}}$$

Where each $i$ is a frame of the video and $x$ and $y$ are horizontal and vertical coordinate locations respectively, in pixels. These values were then summed across all objects from a single replicate to give a total path length for that population. Note that the tracking model struggles to follow individuals moving over each other, therefore an ‘object’ does not correspond to an individual beetle over the length of the recording, but a feature in the recording with locations assigned to it by the tracking model over a series of consecutive frames - as such the number of objects detected in a recording is many more than the number of individual beetles, because many objects may be different components of the same individual’s path.

To account for beetles escaping during the observation period, time of escapes was noted from videos and the sum taken of length of time each beetle was present to give a measure of the number of beetle-seconds represented in each video observation. Total path lengths were divided by beetle-seconds per replicate to give the measure of mean distance traveled per individual per second.

Sinuosity of beetle paths (S; a measure of straightness of travel) was calculated from location data as:

$$S = 2[ P({\frac{{1-c}^{2} {-s}^{2}}{{(1-c)}^{2} + s^{2}}+b^{2})]}^{-0.5}$$

Where P = the physical distance between location readings, b = the coefficient of variation of P, c = the mean sine of all turning angles within a path, s=the mean cosine of all turning angles within a path [(Benhamou, 2004)](https://paperpile.com/c/43JZH5/st1s). Prior to the calculation, object tracks were trimmed to the longest sequence where no location was within 1/35 of the width of the area from any edge - because encountering the edge would influence the path. Object tracks were then ‘rediscretised’ (given a standardised physical distance between recorded locations) of p - this accounts for spatial autocorrelation of turning angles in data with high measurement density (Benhamou 2004). Sinuosity was independently computed for each tracked object and a mean taken across all objects from a single video file.

**Table S1.** Values of parameters used when applying a trained keypoint pose predictor algorithm to track *Tribolium castaneum* beetles from pre-recorded videos in software LOOPY (LoopBio).

| **Parameter** | **Value** |
| --- | --- |
| Score | 0.5 |
| Max detections per class | 10 |
| Min detection distance | 8 |
| Max tracking distance | 22 |
| Death threshold | 10 |


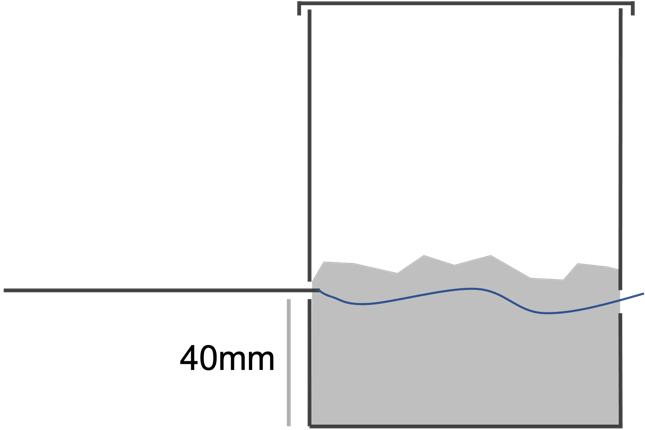


**Figure S3.** Experimental arena used to assay the surface affinity behaviour of experimental *Tribolium castaneum* populations and provide a basis on which to artificially select individuals displaying high and low surface affinity. By pulling a string (blue) threaded through small holes in the walls of the container, a stiff plastic separator could be pulled through a slit in the opposite wall. This completely isolated fodder and beetles within 8mm of the fodder surface from those below.

**Supplementary results**

**Activity and movement pattern**

Sinuosity models using the larger rediscretisation distance (P=20) produced qualitatively the same results as those using P=10, for completeness we give the full results for P=20 here. Beetles from low dispersal lines moved with more sinuous paths than did beetles from high dispersal lines (P=20, β=0.007, SE=0.002, p<0.01), and also mov**ed more sinuously than high dispersal lines (P=20, β=0.005, SE=0.003, p=0.049).** The control treatment sinuosity was intermediate between high and low lines, but did not differ from high lines (P=20, β=-0.002, SE=0.003, p=0.42).

**Morphology**


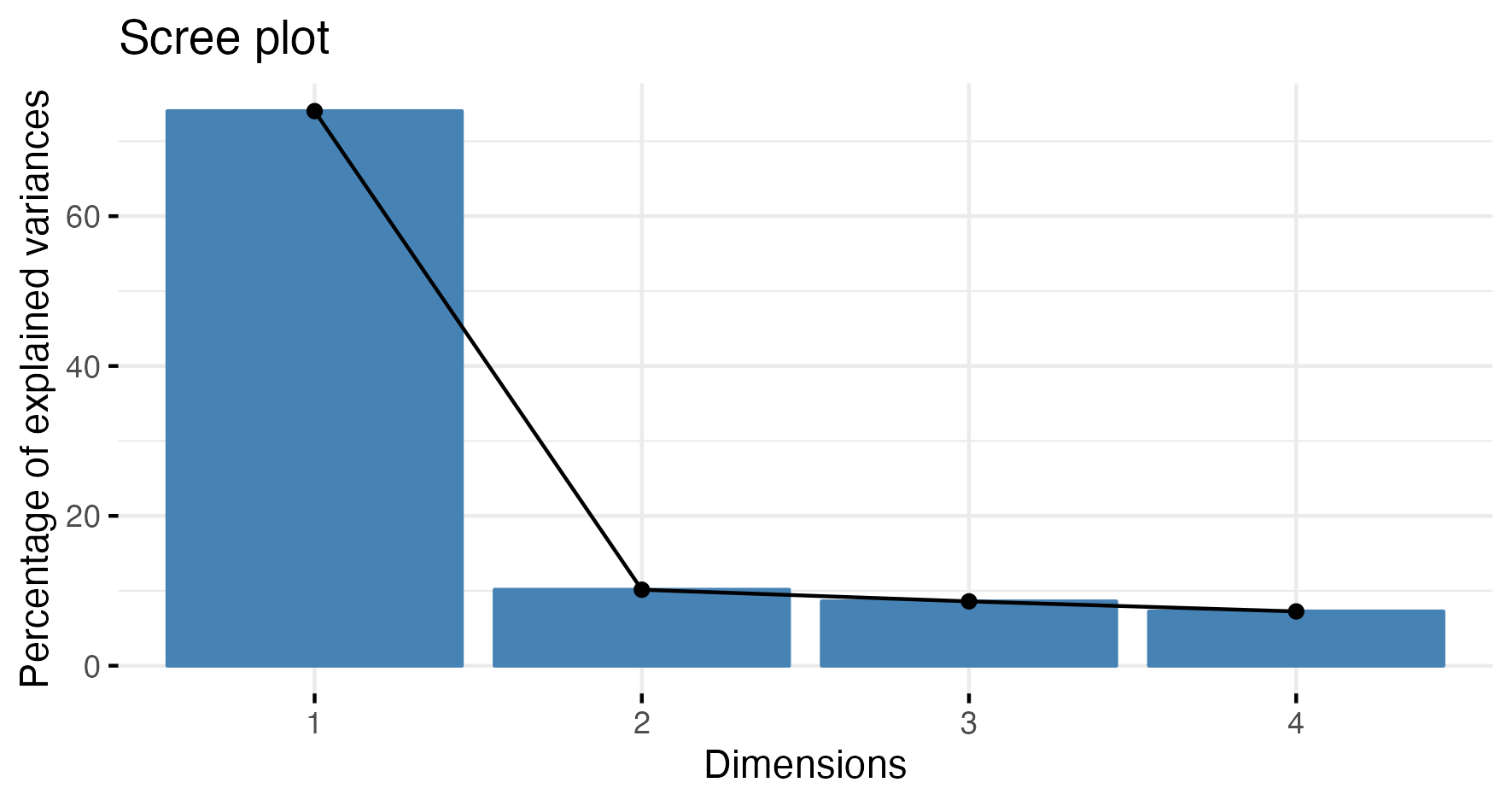


**Figure S4.** The variance explained by each principal component generated by factor analysis on morphological variables measured from *T. castaneum* individuals taken from replicate lines experimentally evolved for high (h), control (no selection; KSS) or low (l) levels of dispersal behaviour.

**Table S2.** Loadings for each principal component generated by factor analysis on morphological variables measured from *T. castaneum* individuals taken from replicate lines experimentally evolved for high (h), control (no selection; KSS) or low (l) levels of dispersal behaviour.

| **Variable** | **PC1** | **PC2** | **PC3** | **PC4** |
| --- | --- | --- | --- | --- |
| Elytra length | -0.5024428 | -0.05924221 | -0.8126212 | 0.2892894 |
| Femur length | -0.5082749 | 0.20898609 | 0.5291783 | 0.6464919 |
| Femur width | -0.4888609 | -0.77011670 | 0.2402095 | -0.3320160 |
| Tibia width | -0.5002229 | 0.59977949 | 0.0437777 | -0.6229969 |

**Table S3.** Repeatability of morphological measurements taken from *Tribolium castaneum* beetles artificially selected for dispersal propensity.

|  |  | **Repeatability (Spearman’s rank correlation)** | |
| --- | --- | --- | --- |
| **Variable** | **N** | **rho** | **p** |
| Elytra length | 90 | 0.99 | <0.001 |
| Femur length | 51 | 0.98 | <0.001 |
| Femur width | 82 | 0.91 | <0.001 |
| Tibia length | 83 | 0.92 | <0.001 |
| First tarsus segment length | 55 | 0.73 | <0.001 |
